# Predictors of Length of Stay, Rehospitalization and Mortality in Community-Acquired Pneumonia Patients: A Retrospective Cohort Study

**DOI:** 10.3390/jcm12175601

**Published:** 2023-08-28

**Authors:** Giorgia Lüthi-Corridori, Maria Boesing, Andrea Roth, Stéphanie Giezendanner, Anne Barbara Leuppi-Taegtmeyer, Philipp Schuetz, Joerg D. Leuppi

**Affiliations:** 1 University Centre of Internal Medicine, Cantonal Hospital Baselland, Rheinstrasse 26, 4410 Liestal, Switzerland; giorgia.luethi-corridori@ksbl.ch (G.L.-C.);; 2 Faculty of Medicine, University of Basel, Klingelbergstrasse 61, 4056 Basel, Switzerland; 3 Department of Patient Safety, University Hospital Basel, Petersgraben 4, 4031 Basel, Switzerland; 4 Cantonal Hospital Aarau, University Department of Medicine, Tellstrasse 25, 5001 Aarau, Switzerland

**Keywords:** community-acquired pneumonia, length of hospital stay, rehospitalization, mortality, prediction, CAP, LOHS

## Abstract

Background: Community-acquired pneumonia (CAP) represents one of the leading causes of hospitalization and has a substantial impact on the financial burden of healthcare. The aim of this study was to identify factors associated with the length of hospital stay (LOHS), rehospitalization and mortality of patients admitted for CAP. Methods: A retrospective cohort study was conducted with patients presenting to a Swiss public hospital between January 2019 and December 2019. Zero-truncated negative binomial and multivariable logistic regression analyses were performed to assess risk factors. Results: A total of 300 patients were analyzed (median 78 years, IQR [67.56, 85.50] and 53% males) with an average LOHS of 7 days (IQR [5.00, 9.00]). Of the 300 patients, 31.6% (97/300) were re-hospitalized within 6 months, 2.7% (8/300) died within 30 days and 11.7% (35/300) died within 1 year. The results showed that sex (IRR = 0.877, 95% CI = 0.776–0.992, *p*-value = 0.036), age (IRR = 1.007, 95% CI = 1.002–1.012, *p*-value = 0.003), qSOFA score (IRR = 1.143, 95% CI = 1.049–1.246, *p*-value = 0.002) and atypical pneumonia (IRR = 1.357, 95% CI = 1.012–1.819, *p*-value = 0.04) were predictive of LOHS. Diabetes (OR = 2.149, 95% CI = 1.104–4.172, *p*-value = 0.024), a higher qSOFA score (OR = 1.958, 95% CI = 1.295–3.002, *p*-value = 0.002) and rehabilitation after discharge (OR = 2.222, 95% CI = 1.017–4.855, *p*-value = 0.044) were associated with a higher chance of being re-hospitalized within 6 months, whereas mortality within 30 days and within one year were both associated with older age (OR = 1.248, 95% CI = 1.056–1.562, *p*-value = 0.026 and OR = 1.073, 95% CI = 1.025–1.132, *p*-value = 0.005, respectively) and the presence of a cancer diagnosis (OR = 32.671, 95% CI = 4.787–369.1, *p*-value = 0.001 and OR = 4.408, 95% CI = 1.680–11.43, *p*-value = 0.002, respectively). Conclusion: This study identified routinely available predictors for LOHS, rehospitalization and mortality in patients with CAP, which may further advance our understanding of CAP and thereby improve patient management, discharge planning and hospital costs.

## 1. Introduction

Community-acquired pneumonia (CAP) is one of the leading causes of hospitalization and is responsible for approximately 2.5 million deaths worldwide every year [1,2]. In Europe, CAP also leads to high hospitalization rates, causing a significant financial burden for the healthcare system [3,4]. The financial impacts of CAP due to prolonged hospitalizations or increased hospitalization rates have been documented in previous studies [5,6,7]. Current guidelines emphasize the importance of discharging patients as soon as they achieve clinical stability and have access to a safe environment where continuity of care can be ensured [8]. The recommendations particularly underline the importance of increasing outpatient treatment to decrease the cost of hospitalizations and the risk of hospital-acquired complications [8]. However, the length of hospital stay (LOHS) for patients with CAP continues to be variable and for that reason, the development of accurate models to predict the LOHS using patients’ baseline profiles from an early stage is needed. Obtaining accurate predictive models upon admission has multiple advantages. First of all, they allow us to identify the profiles of patients at risk of prolonged hospitalization, and whenever possible, to promptly act on modifiable factors. Moreover, discharge strategies can be improved. The implementation of a precise prediction model would additionally permit the evaluation of hospital performance, thereby fostering advancements in hospital management.

The LOHS in patients with CAP can be influenced by a variety of factors, including sociodemographic, health-related and hospital care-related characteristics [9,10,11,12,13,14,15,16,17,18,19,20,21]. A number of previous studies investigating factors that influence the LOHS in CAP identified patient-related variables such as advanced age and specific comorbidities, in addition to disease severity, as predictors of a prolonged LOHS [9,10,11,12,13]. Other studies direct their research focus to laboratory values [14,15,16], while others concentrate on therapies [17,18,19] or other interventions during hospitalization [20,21]. Due the wide variety of influencing factors, there is no uniform method for predicting the LOHS in CAP patients; moreover, as mentioned above, several studies included factors that are not available at the time of admission, hindering the chance of predicting the LOHS in the first days of hospitalization.

The primary aim of this study was to identify which factors may affect the length of stay of patients admitted for CAP. The identification of patient characteristics influencing the LOHS may help decision makers properly plan hospital management. Particularly, we retrospectively explored whether the primary outcome, the LOHS for CAP, was associated with commonly available sociodemographic and health-related variables that are measurable at the time of admission to the hospital.

Despite advances in therapy, the mortality rate associated with this disease is still high (6–10%). While a shorter LOHS may decrease hospital costs, it may also negatively impact the quality of care [22]. Moreover, research has indicated that rehospitalization and mortality rates are high among patients with CAP who survive the initial admission. This is primarily attributed to factors related to the aging population, like the presence of multiple medical conditions and other health fragilities [23]. Most elderly CAP patients require special attention from health care professionals after discharge to reduce rehospitalization and mortality rates [24]. For this reason, this study analyzed factors associated with rehospitalization within 6 months and all-cause mortality (30-day and one-year mortalities) as secondary outcomes.

## 2. Materials and Methods

### 2.1. Design and Setting

Our study was conducted in the cantonal hospital of Baselland (KSBL), a district general hospital covering a stable population of 280,000 in Northwest Switzerland. We undertook a retrospective cohort study extracting all patients older than 18 years of age who were admitted to the hospital between January and December 2019 and categorized them using an International Classification of Disease (ICD) code related to pneumonia (for more details, see the ICD codes list in the Appendix A). A total of 573 patients were identified.

### 2.2. Inclusion and Exclusion Criteria

Cases were included in this study if newly diagnosed CAP was the main reason for the patient’s hospitalization and their diagnosis was confirmed via a chest X-ray or a microbiological test supported by clinical judgment. CAP was defined according to the Infectious Diseases Society of America (IDSA) criteria [8].

The following criteria were applied for exclusion:

- Denied research consent (n = 31);
- Hospital-acquired pneumonia (n = 26);
- Immunocompromised patients (n = 35);
- Patients with prior therapy prescribed by their general practitioner, not newly diagnosed nor newly treated (n = 83);
- Diagnosis not confirmed (n = 38);
- Directly transferred to rehabilitation (n = 27);
- Palliative care (n = 15);
- Other main diagnosis or main reason for hospitalization (n = 11);
- Consecutive (second or third) admission for CAP in the study period (n = 7).

**Figure 1:**
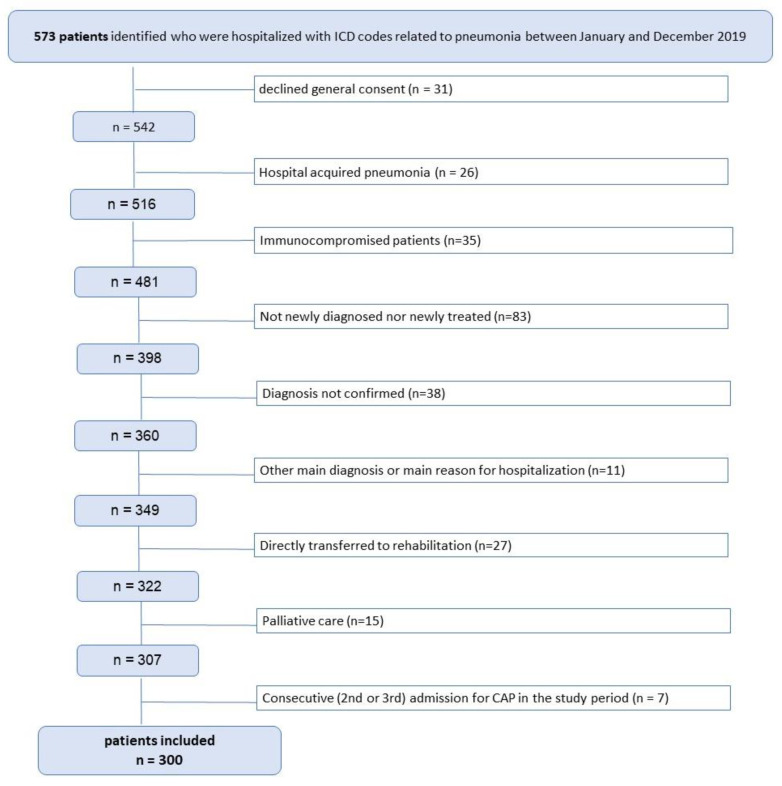
Flowchart diagram for the patient selection process.

After the application of the eligibility criteria, the data of 300 patients were included in the analysis (Figure 1).

### 2.3. Data Collection

Basic data such as gender, age and the LOHS were automatically extracted from the controlling system. The remaining variables were extracted manually from the electronic patient record by a study physician. To ensure the quality of the data, a subset was reviewed by a health scientist. The primary outcome of interest was the LOHS. Additionally, the secondary outcomes included rehospitalization within six months and all-cause mortality within 30 days and one year. To minimize the risk of bias, optimism and overfitting, we did not perform a data-driven selection of variables. Instead, potential predictors were selected based on the existing literature and clinical knowledge. Two researchers conducted a comprehensive literature review and consulted clinical experts in the field. Predictors for the LOHS included variables available at the time of admission: demographic variables, vital signs, laboratory parameters, comorbidities and risk scores. An “Indication for oxygen supplementation” was defined as the presence of at least one of the following conditions upon admission: oxygen saturation < 90%, oxygen supplementation already in place and respiratory rate ≥ 30. For the analysis of the rehospitalization rate and mortality, events occurring during the hospitalization were also collected, such as oxygen supplementation during hospitalization and rehabilitation after discharge.

### 2.4. Statistical Analyses

The outcome variables comprised the LOHS (primary outcome), all-cause mortality (at 30 days and 1 year) and rehospitalization within 6 months (secondary outcomes).To minimize the risk of bias, optimism and overfitting, no data-driven selection of variables was conducted. The parameters assessed included age, gender, housing situation before admission, type of pneumonia (atypical pneumonia when an atypical pathogen was identified), medical history and vital signs obtained at the time of admission, laboratory results, therapy and diagnostic work-up score. The analysis of the LOHS was primarily conducted on patients that were discharged alive; since only one patient died in hospital, it was not necessary to perform a sensitivity analysis on the full data set. For the re- hospitalization outcome, we further included the variable LOHS into the model, rehabilitation after discharge and oxygen supplementation during hospitalization. The selection criteria for a multivariate regression of mortality and rehospitalization were tailored to the specific nature of the outcomes under investigation. Distinct from the LOHS analysis, which included admission-time variables like vital signs, the multivariable regression for rehospitalization and mortality focused on long-term outcomes (6 month and 1 year, respectively), retaining factors with minimal temporal variability such as demographics, comorbidities and hospital-related factors (e.g., oxygen during hospitalization, the LOHS and post-discharge rehabilitation) to minimize the risk of susceptibility to temporal fluctuations. We displayed measures of central tendency for descriptive statistics: a median with an interquartile range (IQR) if the distribution was skewed (as determined via a histogram assessment). For categorical variables, we reported absolute and relative frequencies. Variables with missing values of up to 30% were imputed using the k-nearest neighbor algorithm (function knn.impute from the R package “bnstruct”) [25,26]. A zero-truncated negative binomial regression was conducted to estimate the LOHS and its association with potential risk factors using the R package “VGAM”. Logistic regression models were created to estimate the risk of death and rehospitalization and its association with potential risk factors using the R package “stats”. All statistical analyses were performed using R version 4.0.3 statistical software (R Foundation for Statistical Computing). All reported *p*-values were two-sided; statistical significance was defined as *p* < 0.05.

## 3. Results

### 3.1. Patient Characteristics

The patient characteristics are presented in Table 1. The median age at the time of hospital admission was 78.5 years, and 53% were males. More than half of the patients had chronic cardiovascular comorbidities (58%); the second most frequent concomitant disease was COPD, followed by diabetes (29.7% and 18.3%, respectively).

### 3.2. Prediction of the LOHS, Rehospitalization and Mortality

Our primary aim was to examine the factors associated with the LOHS. Table 2 provides coefficient estimates for the predictors of the LOHS in patients who did not die. Regression coefficients are shown as incident risk ratios (IRRs). The median LOHS of the overall cohort was 7 days. The analysis of the prediction model for the LOHS identified four statistically significant predictors: sex, age, qSOFA score and atypical pneumonia. The LOHS prediction at the intercept (7.5 days) is the LOHS when all covariates are at 0 (for categorical covariates) or at their mean (for continuous covariates). The predicted LOHS of the model for each variable is presented for a one-unit increase. A higher increase occurs when the qSOFA score increases the predicted LOHS rise by one unit to 8.5 days. Women tended to stay one night longer than men, while people with atypical pneumonia compared to those without tended to stay three nights longer, assuming all other variables are held constant.

Our secondary aims included the analyses of factors associated with rehospitalization and mortality. The results for our secondary outcome concerning the rehospitalization rate are reported in Table 3. The odds for rehospitalization within 6 months in the KSBL were also significantly higher for patients with a higher qSOFA score at admission. Moreover, patients with diabetes and those who were admitted to rehabilitation had a higher chance of being rehospitalized within 6 months. No other variable was found to be significantly associated with rehospitalization.

The results of the multivariable logistic regression models for mortality are displayed in Table 4 (30-day mortality) and Table 5 (1-year mortality). In both predictive models, age and an active cancer diagnosis were the only two significant variables associated with mortality. No other variable was found to be significantly associated with mortality.

## 4. Discussion

This retrospective observational cohort study of patients with CAP showed that the LOHS is influenced by demographic factors such as an older age and female gender and by disease-specific factors like the qSOFA score and atypical pneumonia. Other factors, such as other types of comorbidities, vital signs (other than included in the qSOFA) and laboratory values at admission, were not associated with a longer LOHS.

Interestingly, our results show that women had worse outcomes compared to men. Gender differences have been observed in the clinical course, and outcomes of people with CAP and, historically, men have been found to have worse outcomes, particularly in terms of short- and long-term mortality [27,28]. Although little evidence in terms of the LOHS is available, our results are consistent with an international multicenter study by the Community Acquired Pneumonia Organization which followed patients for 10 years. In this study, Arnold and colleagues found that women had significantly longer LOHSs and also worse outcomes in terms of time until clinical stability and mortality within 28 days [29]. Gender differences clearly warrant further confirmation and validation because causal inference cannot be drawn. However, if confirmed in the future, the current concept that female patients have a lower risk than males with CAP may need to be revised and the current scoring system adjusted (for example, the subtraction of 10 points for females in the Pneumonia Severity Index (PSI)).

The quick Sequential (Sepsis-related) Organ Failure Assessment (qSOFA) score is another severity assessment tool and validated prognostic model devised by Seymour et al. [30,31] Originally it was developed to predict sepsis using three main clinical criteria, namely altered mental status, low systolic blood pressure and high respiratory rate. In line with other studies, our results also confirm the prognostic validity of the qSOFA score in predicting the length of hospital stay [30,32,33,34]. The role of qSOFA in the LOHS was confirmed recently by Koch et al. [35]; however, the impacts of the single items comprising the score were unclear. For this reason, in our study, we also analyzed the items of the qSOFA score separately, and we found that altered mental status (GCS < 15) and blood pressure (Systolic BP ≤ 100) were significantly predictive for the LOHS (for more details, see Table A1 in Appendix A). The main advantage of implementing the qSOFA score is that it does not require laboratory tests and allows for rapid and repetitive assessments. In addition to the task force’s recommendation to use the qSOFA tool to further investigate potential organ dysfunction or to initiate or escalate appropriate therapy, our results suggest that the qSOFA score can be integrated into predictive models as a risk predictor for an extended LOHS.

Another point worth discussing is the fact that atypical pneumonia was predictive for an extended LOHS. In community-acquired pneumonia, examples of typical pathogens are streptococcus pneumoniae and haemophilus influenzae, and atypical pathogens are mycoplasma pneumoniae, chlamydia pneumoniae and staphylococcus aureus [36]. Atypical pneumonia often expresses more unspecific symptoms such as headache, low fever, dyspnea, dry cough and only slightly elevated inflammatory biomarkers; moreover, the clinical presentation can range from mild symptoms to severe illness with respiratory failure or sepsis [37]. Approximately 7% to 20% of cases of community-acquired pneumonia are believed to be caused by atypical bacterial microorganisms which cannot be detected via Gram staining and pose challenges in terms of culturing [38]. Moreover, the presence of atypical pathogens in some patients with community-acquired pneumonia (CAP) poses a challenge in the selection of empirical antibiotic treatment. These pathogens are inherently resistant to beta-lactam drugs, which are commonly used as an initial antibiotic treatment [39]. This dilemma arises from the fact that adding antibiotic coverage specifically for atypical pathogens might carry the risk of adverse effects and promotes the development of antimicrobial resistance [40]. On the other hand, withholding such coverage may potentially worsen the prognosis if an atypical pathogen is indeed the causative agent of the pneumonia [41,42]. Therefore, in our study, we also considered the presence of atypical pathogens as a potential predictor when examining the length of stay in patients with CAP. We recognized that the use or omission of antibiotic coverage for atypical pathogens could influence the clinical course and outcomes, including the LOHS. Hence, the observed association between atypical pneumonia and an extended length of stay in our study could potentially be attributed to the challenges involved in treatment. Specifically, the addition of antibiotic treatment coverage to address atypical pathogens might inadvertently lead to adverse effects, thereby prolonging the hospitalization period. Alternatively, the diagnostic tests employed to identify atypical pathogens may require additional time, contributing to a longer length of stay.

Our secondary outcomes included rehospitalization in the KSBL within 6 months. We detected that in our study population, rehospitalization within 6 months was significantly associated with factors such as diabetes, qSOFA score and rehabilitation after discharge. The percentage of patients who were rehospitalized within 6 months was 31.6%, which is similar to the ranges of two non-recent studies in which the assessed cumulative readmission rates were 22 and 35.6% [43,44]. In terms of readmission rate, in fact, it is not common to assess long-term outcomes, as stated by Prescott in a systematic review; the majority of published studies in the literature concentrate their focus on the 30-day readmission, and the percentage varies from a minimum of 16.8 to a maximum of 20.1% [45]. The most recent study published in 2021 by Averin et al., which assessed late readmission following hospitalization for pneumonia among American adults, analyzed one-year readmission, and the proportion reached 42.3% of the study population [46]. As previously mentioned the qSOFA score is a validated prognostic tool for sepsis; a recent study investigated the prognostic performance of the qSOFA score for in-hospital mortality and ICU admission [47], but its accuracy in predicting long-term outcome in terms of rehospitalization within 6 months has not been established.

Interestingly, diabetes was the only chronic health condition associated with rehospitalization within 6 months. Previous studies found a relationship between diabetes and the incidence of CAP [48] or hospitalization rate [49] or demonstrated that patients with diabetes have worse discharge outcomes compared to patients without diabetes [50]. A recently published systematic review and meta-analysis by Fang et al. found that diabetes mellitus was significantly associated with the hospital readmission rate among pneumonia patients (pooled OR = 1.18; 95% CI: 1.08–1.28) [24], which is confirmed by our results. So, despite advances in treatment, diabetes is still associated with a higher risk of adverse outcomes, and healthcare providers should take this finding into account. Although CAP patients who also suffer from diabetes are at an elevated risk for adverse events and a complicated clinical course, as explained above, further studies are required in order to clarify the underlying mechanisms and the impact of a disrupted glucose metabolism on the development and clinical outcome of CAP in light of rehospitalization rates.

It is worth mentioning that discharge into rehabilitation was found to be significantly associated with rehospitalization. Patients who were sent to rehabilitation after discharge had a higher chance of being readmitted to the hospital within 6 months compared to those who did not attend rehabilitation. This finding contradicts the initial hypothesis that rehabilitation would reduce the risk of rehospitalization. Possible explanations may include the complexity and severity of the underlying conditions requiring rehabilitation, the intensity or duration of the rehabilitation program, or other unmeasured factors that could influence the outcome. In order to further investigate the underlying reasons for the positive relationship between rehabilitation and rehospitalization, we conducted a post hoc analysis comparing the characteristics of patients who were rehabilitated after hospitalization with those who were not rehabilitated. As displayed in Table A2 in Appendix A, significant differences were detected. The age of patients who received rehabilitation was significantly higher compared to those who did not (medians of 82.73 and 77.35, respectively; *p*-value = 0.004). Similarly, patients who underwent rehabilitation had a significantly longer LOHS (medians of 11 days and 6 days, respectively 6.00; *p*-value = 0.001). Other factors, such as chronic cardiovascular disease, COPD, respiratory insufficiency, parapneumonic effusion and cardiovascular complications, also showed significant differences between the two groups. The detected significant differences between the two groups in terms of age, comorbidity burden and hospital complications might explain the positive association between rehabilitation and rehospitalization. Hence, it is necessary to carefully interpret the association between rehabilitation and rehospitalization, considering the confounding effects of these patient characteristics. Moreover, a previous study showed promising results, especially in the short-term, specifically focusing on the 30-day hospital readmission rate [51]. The majority of the studies investigating the positive effects of rehabilitation mainly focused on different outcomes [52,53,54,55]. It is important to note that our study differs from these previous investigations as we examined rehospitalization rates within a longer time frame of six months. This extended duration allowed us to capture readmissions that may have occurred beyond the initial 30-day period and provides a more comprehensive understanding of the factors influencing rehospitalization. Further exploration is needed to better understand this unexpected association.

In terms of mortality, we observed that the in-hospital mortality rate was very low: only one patient died during the initial hospitalization, as displayed in Table 1. This can be explained by the fact that all the patients who were transferred for palliative care or directly sent to another hospital were excluded from this study. On the contrary, we noticed that almost one-quarter of the overall mortality within one year happened within thirty days after discharge (22.9%). This trend was also confirmed by Wadhera in a study using population-based data from almost 16300 patients which was conducted in Germany. The research revealed a significant increase in mortality over time, with a 4.7% increase between in-hospital mortality (17.2%) and 30-day mortality (21.9%) [56]. Similarly, a study conducted in the United States with a 10-year cohort of about 3 million CAP patients reported a high 30-day post-discharge mortality of 8.2% [57]. Both multivariable logistic models for 30-day and 1-year mortality revealed that age and a cancer diagnosis were associated with a higher risk of mortality. The findings from our study reinforce prior observations that all-cause mortality during the year subsequent to hospital admission for pneumonia is linked to increasing age and a worsening comorbidity profile [46,58,59]. A recent study concluded that while long-term mortality following CAP was primarily associated with comorbidities, there is potential for early post-discharge complications (within 30 days) to be attributed to CAP-related issues that may benefit from targeted interventions [60]. However, our results did not find different predictors between the two mortality outcomes. Finally, it is important to note that the LOHS was not significantly associated with mortality nor rehospitalization, implying that a shorter LOHS did not show an increased risk of re-admission or post-discharge mortality.

### Strengths, Limitations and Further Research

The novelty of our study lies in its comprehensive encompassing of three important quality indicators as research outcomes (the LOHS, rehospitalization and mortality). The prediction models included various factors such as demographic variables, health-related variables and laboratory values available at the time of admission. A further strength of our research was the possibility to investigate long-term outcomes such as mortality within one year, as these data were available for all patients. However, there are certain limitations to consider. As a retrospective study, the quality of the data depended on accurate documentation in the patient files, which may have resulted in incomplete information. It is especially important to note that the presentation of the severity index data, such as the Pneumonia Severity Index (PSI), was hindered by the absence of available data, thereby limiting the depth of the analysis regarding the severity stratification of CAP cases in this study. Furthermore, information on rehospitalization within six months was limited to a specific hospital due to privacy policies, potentially missing readmissions to other healthcare facilities. However, according to a previous study, in Switzerland, most unplanned readmissions occur within the same hospital [61]. The conclusions of this study are limited to the definition of CAP according to the IDSA criteria [8]. The generalizability of other definitions of CAP will have to be assessed. Overall, our study provides a foundation for future research and contributes valuable insights into other aspects of CAP, particularly focusing on the possible predictors of the LOHS, mortality and rehospitalization that are available at the time of admission. The identification of predictors available at the time of admission might help to promptly identify patients who are at a higher risk of adverse outcomes and allow healthcare providers to prioritize their care, allocate appropriate resources and develop personalized management strategies tailored to patients’ specific needs. Further studies are needed to investigate the underlying causes contributing to the association between atypical pneumonia and the LOHS. As mentioned before, predictive models could include data regarding antibiotic coverage and time until the diagnosis of atypical pneumonia. By conducting additional research, a more comprehensive understanding can be obtained, and targeted interventions to optimize patient care and reduce the burden associated with prolonged hospital stays can be developed.

## 5. Conclusions

Understanding the factors that are associated with the LOHS in patients with CAP has clinical implications and may help healthcare providers to deliver efficient care and allocate adequate resources in the management of these patients. In summary, the results of this study showed that female sex, advanced age, a higher qSOFA score and atypical pneumonia were predictive for a longer LOHS. Diabetes, a high qSOFA score and discharge to rehabilitation were associated with a higher chance of rehospitalization within 6 months, whereas mortality rates within 30 days and within one year were both linked to advanced age and the presence of an active cancer diagnosis. However, the potential unfavorable effect of rehabilitation after hospitalization should be interpreted with caution as a post hoc analysis revealed significant disparities in terms of age, LOHS, comorbidities and hospital complications among the studied groups (patients undergoing rehabilitation after hospitalization and those not being rehabilitated). Moreover, our study confirmed the important role of the qSOFA score as a predictive tool not only for sepsis but also for the LOHS and rehospitalization in patients with CAP.

## Figures and Tables

**Table 1:** Patient characteristics.

	All (n = 300)	Missing n (%)
**Demographic**		
Age at diagnosis, median [IQR]	78.48 [67.56, 85.50]	--
Gender (males), n (%)	160/300 (53.3%)	--
**Vital Signs**		
Respiratory rate at admission, median [IQR]	21.00 [18.00, 26.00]	77 (25.7%)
Indication for oxygen supplementation, n (%)	102/298 (34.2%)	2 (0.7%)
Oxygen supplementation during hospitalization, n (%)	135/299 (45.2%)	1 (0.3%)
Body temperature at admission, median [IQR]	37.60 [36.95, 38.40]	9 (3.0%)
Fever at admission, n (%)	114/293 (38.9%)	7 (2.3%)
Heart rate at admission, median [IQR])	91.00 [79.00, 104.00]	1 (0.3%)
Systolic blood pressure at admission, median [IQR]	132.00 [112.50, 147.00]	1 (0.3%)
Diastolic blood pressure at admission, median [IQR]	74.00 [65.00, 85.00]	1 (0.3%)
**Comorbidities**		--
Chronic cardiovascular, n (%)	174/300 (58.0)	--
Hypertension, n (%)	177/300 (59.0%)	--
Cancer, n (%)	32/300 (10.7%)	--
Diabetes, n (%)	55/300 (18.3%)	--
Asthma, n (%)	22/300 (7.3%)	--
COPD, n (%)	59/300 (19.7%)	--
Other chronic respiratory diseases, n (%)	42/300 (14.0%)	--
**Risk Scores**		
GCS at admission, median [IQR]	15.00 [15.00, 15.00]	7 (2.3%)
qSOFA, median [IQR]	1.00 [0.00, 1.00]	82 (27.3%)
BMI, median [IQR]	26.00 [22.40, 30.25]	137 (45.7%)
**Laboratory Values**		
Leucocytes at admission, median [IQR]	11.90 [8.97, 15.00]	--
Atypic pneumonia diagnosed, n (%)	19/300 (6.3%)	--
**Discharge circumstances**		
Rehabilitation, n (%)	51/300 (16.6)	--
Discharged home, n (%)	217/300 (72.3)	--
Discharged to a care facility, n (%)		
**Outcomes**		
LOHS, median [IQR]	7.00 [5.00, 9.00]	--
Rehospitalization within six months, n (%)	97/300 (31.6%)	--
In-hospital death, n (%)	1/300 (0.3%)	--
30-day mortality, n (%)	8/300 (2.7%)	--
1-year mortality, n (%)	35/300 (11.7%)	--

IQR = interquartile range; COPD = chronic obstructive pulmonary disease; GCS = Glasgow coma scale; qSOFA = quick SOFA; BMI = body mass index; LOHS = length of hospital stay.

**Table 2:** Results of multivariable zero-truncated negative binomial regression model for length of hospital stay (LOHS) estimation in CAP patients who survived the first hospital admission (n = 299).

	LOHS Prediction	IRR	(95% CI)	*p*-Value
(Intercept)	7.458	11.947	1.18–121.0	0.036
Gender (males)	6.562	0.877	0.776–0.992	0.036
Age	7.511	1.007	1.002–1.012	0.003
Chronic cardiovascular	8.217	1.103	0.957–1.273	0.176
COPD	7.126	0.955	0.822–1.108	0.542
Asthma	6.654	0.89	0.708–1.119	0.318
Diabetes	7.148	0.958	0.821–1.118	0.583
Active cancer	8.12	1.09	0.905–1.314	0.364
qSOFA	8.508	1.143	1.049–1.246	0.002
Heart rate at admission	7.472	1.002	0.999–1.005	0.218
Body temperature at admission	7.207	0.966	0.909–1.026	0.26
CRP at admission	7.459	1	1–1.001	0.631
Leucocytes at admission	7.46	1	0.993–1.008	0.936
Atypic pneumonia diagnosed	10.088	1.357	1.012–1.819	0.041

LOHS = length of hospital stay; IRR = incidence rate ratio; CI = confidence interval; COPD = chronic obstructive pulmonary disease; qSOFA = Quick SOFA; CRP = C-reactive Protein.

**Table 3:** Results of multivariable logistic regression model for rehospitalization within 6 months in patients with CAP.

	OR	(95% CI)	*p*-Value
Gender (males)	0.964	0.549–1.693	0.898
Age	1.016	0.994–1.039	0.164
Chronic cardiovascular	1.123	0.582–2.177	0.73
COPD	1.021	0.504–2.016	0.954
Asthma	1.759	0.622–4.718	0.269
Diabetes	2.149	1.104–4.172	0.024
Active cancer	1.565	0.682–3.557	0.284
qSOFA	1.958	1.295–3.002	0.002
Oxygen during hospitalization	0.636	0.357–1.116	0.118
LOHS	1.055	0.984–1.132	0.134
Rehabilitation after discharge	2.222	1.017–4.855	0.044

OR = odds ratio; CI = confidence interval; COPD = chronic obstructive pulmonary disease; qSOFA = quick SOFA; LOHS = length of hospital stay.

**Table 4:** Results of multivariable logistic regression model for 30-day mortality in patients with CAP.

	OR	(95% CI)	*p*-Value
Gender (males)	13.219	1.235–483.5	0.075
Age	1.248	1.056–1.562	0.026
Chronic cardiovascular	0.953	0.078–25.31	0.972
COPD	0.335	0.012–3.534	0.419
Asthma	0	0–0	0.993
Diabetes	0.956	0.059–9.993	0.971
Active cancer	32.671	4.787–369.1	0.001
qSOFA	0.817	0.198–3.135	0.768
Oxygen during hospitalization	6.787	0.864–101.4	0.103
LOHS	1.144	0.909–1.447	0.246
Rehabilitation after discharge	0.259	0.010–3.353	0.356

OR = odds ratio; CI = confidence interval; COPD = chronic obstructive pulmonary disease; qSOFA = auick SOFA; LOHS = length of hospital stay.

**Table 5:** Results of multivariable logistic regression model for one-year mortality in patients with CAP.

	OR	(95% CI)	*p*-Value
Gender (males)	1.352	0.594–3.166	0.477
Age	1.073	1.025–1.132	0.005
Chronic cardiovascular	1.53	0.563–4.684	0.425
COPD	0.722	0.247–1.881	0.525
Asthma	0.773	0.106–3.445	0.763
Diabetes	1.847	0.725–4.518	0.185
Active cancer	4.408	1.680–11.43	0.002
qSOFA	1.194	0.665–2.126	0.547
Oxygen during hospitalization	1.6	0.714–3.657	0.256
LOHS	1.025	0.922–1.130	0.63
Rehabilitation after discharge	1.234	0.401–3.541	0.703

OR = odds ratio; CI = confidence interval; COPD = chronic obstructive pulmonary disease; qSOFA = quick SOFA; LOHS = length of hospital stay.

## Data Availability

All data generated were analyzed during this study and the results are included in this article. The data presented in this study are available upon reasonable request from the corresponding author. The data are not publicly available due to restrictions on data privacy.

## Appendix A

A list of the ICD-10 Codes used for patients’ selection, in detail:

− A 48.1 Pneumonic legionnaires disease;
− J 10.0 Influenza due to other identified influenza virus with unspecified type of pneumonia;
− J 12.0 Adenoviral pneumonia;
− J 12.1 Respiratory syncytial virus pneumonia;
− J 12.2 Parainfluenza virus pneumonia;
− J 12.3 Human metapneumovirus pneumonia;
− J 12.8 Other viral pneumonia;
− J 12.9 Viral pneumonia, unspecified;
− J 13 Pneumonia due to Streptococcus pneumoniae;
− J 14 Pneumonia due to Hemophilus influenzae;
− J. 15.1 Pneumonia due to Pseudomonas;
− J 15.2 Pneumonia due to Staphylococcus;
− J 15.3 Pneumonia due to streptococcus, group B;
− J 15.4 Pneumonia due to other streptococci;
− J 15.5 Pneumonia due to Escherichia coli;
− J 15.6 Pneumonia due to other aerobic Gram-negative bacteria;
− J 15.7 Pneumonia due to Mycoplasma pneumoniae;
− J 15.8 Other bacterial pneumonia;
− J 15.9 Unspecified bacterial pneumonia;
− J 16.0 Chlamydial pneumonia;
− J 16.8 Pneumonia due to other specified infectious organisms;
− J 18.0-Bronchopneumonia, unspecified organism;
− J 18.1 Lobar pneumonia, unspecified organism;
− J 18.8 Other pneumonia, unspecified organism;
− J 18.9 Pneumonia, unspecified organism;
− J 85.1 Abscess of lung with pneumonia.

**Table A1:** Multivariable zero-truncated negative binomial regression model for LOHS estimation in CAP survivors; qSOFA items separately assessed (n = 299).

	LOHS Prediction	IRR	(95% CI)	*p*-Value
(Intercept)	6.693			
Gender (males)	5.883	0.875	0.775–0.989	0.032
Age	6.738	1.007	1.002–1.012	0.005
Chronic cardiovascular	7.455	1.117	0.970–1.285	0.124
COPD	6.455	0.964	0.831–1.117	0.623
Asthma	5.96	0.887	0.708–1.112	0.299
Diabetes	6.49	0.969	0.830–1.131	0.689
Active cancer	7.463	1.118	0.928–1.347	0.241
Altered mental status (GCS < 15)	8.234	1.235	1.079–1.414	0.002
Systolic BP ≤ 100	8.646	1.297	1.064–1.582	0.010
Respiratory rate ≥ 22	7.142	1.069	0.947–1.206	0.282
Heart rate at admission	6.708	1.002	0.999–1.006	0.131
Body temperature at admission	6.478	0.967	0.911–1.027	0.274
CRP at admission	6.694	1	1–1.001	0.458
Leucocytes at admission	6.696	1.001	0.993–1.008	0.877
Atypic pneumonia diagnosed	9.251	1.389	1.039–1.856	0.026

LOHS = length of hospital stay, IRR = incidence rate ratio; CI = confidence interval; COPD = chronic obstructive pulmonary disease; GCS = Glasgow coma scale; BP = blood pressure, CRP = C-reactive protein.

**Table A2:** Post hoc analysis. Comparison between patients undergoing rehabilitation after hospitalization and those not being rehabilitated.

Variables	Overall (n = 300)	Rehabilitation (No) n = 253	Rehabilitation (Yes) n = 47	*p*-Value	Missing
Age, median [IQR]	78.48 [67.6, 85.5]	77.35 [66.2, 84.6]	82.73 [78.0, 88.5]	0.004	0
LOHS, median [IQR]	7.00 [5.00, 9.00]	6.00 [5.00, 8.00]	11.00 [7.5, 14.5]	<0.001	0
BMI (median [IQR])	26.00 [22.4, 30.2]	26.50 [22.8, 30.4]	25.00 [21.0, 27.3]	0.085	45.7
Oxygen during hospitalization, in days, median [IQR]	1.00 [0.0, 3.0]	1.00 [0.0, 3.0]	3.50 [1.0, 6.7]	0.001	26.3
qSOFA, median [IQR]	1.00 [0.00, 1.00]	1.00 [0.0, 1.0]	1.00 [0.0, 1.0]	0.265	27.3
Gender (male), n (%)	160 (53.3)	138 (54.5)	22 (46.8)	0.414	0
Atypic pneumonia, n (%)	12 (4.0)	10 (4.0)	2 (4.3)	1	0
Chronic cardiovascular disease, n (%)	174 (58.0)	140 (55.3)	34 (72.3)	0.045	0
Diabetes, n (%)	55 (18.3)	49 (19.4)	6 (12.8)	0.385	0
COPD, n (%)	59 (19.7)	44 (17.4)	15 (31.9)	0.036	0
Asthma, n (%)	22 (7.3)	21 (8.3)	1 (2.1)	0.236	0
Other chronic respiratory disease, n (%)	42 (14.0)	31 (12.3)	11 (23.4)	0.073	0
Active cancer, n (%)	32 (10.7)	24 (9.5)	8 (17.0)	0.201	0
Severe immunosuppression, n (%)	2 (0.7)	1 (0.4)	1 (2.1)	0.716	0
Care facility resident, n (%)	50 (16.7)	46 (18.2)	4 (8.5)	0.155	0
Oxygen during hospitalization, n (%)	135 (45.2)	106 (42.1)	29 (61.7)	0.02	0.3
Admission to ICU, n (%)	27 (9.0)	20 (7.9)	7 (14.9)	0.208	0
ARDS, n (%)	300 (100.0)	253 (100.0)	47 (100.0)	NA	0
Sepsis, n (%)	6 (2.0)	4 (1.6)	2 (4.3)	0.525	0
Respiratory insufficiency, n (%)	25 (8.3)	15 (5.9)	10 (21.3)	0.001	0
Cardiovascular complications, n (%)	42 (14.0)	29 (11.5)	13 (27.7)	0.007	0
Acute kidney injury, n (%)	54 (18.0)	45 (17.8)	9 (19.1)	0.987	0
Anemia, n (%)	19 (6.4)	19 (7.6)	0 (0.0)	0.109	1
Parapneumonic effusion, n (%)	34 (11.3)	22 (8.7)	12 (25.5)	0.002	0
Syncope, n (%)	3 (1.0)	3 (1.2)	0 (0.0)	1	0
In-hospital fall, n (%)	22 (7.4)	16 (6.3)	6 (12.8)	0.214	0.3
Elevated liver parameters, n (%)	23 (7.7)	21 (8.3)	2 (4.3)	0.51	0
Neurological complications, n (%)	14 (4.7)	9 (3.6)	5 (10.6)	0.082	0
Gastrointestinal complications, n (%)	14 (4.7)	12 (4.7)	2 (4.3)	1	0
Electrolyte disorder, n (%)	53 (17.7)	44 (17.4)	9 (19.1)	0.935	0

IQR = interquartile range; LOHS = length of hospital stay; BMI = body mass index; qSOFA = quick SOFA; COPD = chronic obstructive pulmonary disease; ICU = Intensive care unit; ARDS = acute respiratory distress syndrome.
